# Reliability prediction and evaluation of communication base stations in earthquake prone areas

**DOI:** 10.1038/s41598-023-35841-x

**Published:** 2023-06-02

**Authors:** Xueming Li, Yao Wei, Zheng Ming, Hao Cong, Xuanyu Zheng, Qihai Chang

**Affiliations:** 1 College of Information Engineering, Xizang Minzu University, Xianyang, 712082 China; 2 College of Mathematics and Physics, Chengdu University of Technology, Chengdu, 610059 China; 3 Geomathematics Key Laboratory of Sichuan Province, Chengdu University of Technology, Chengdu, 610059 China

**Keywords:** Natural hazards, Engineering, Mathematics and computing

## Abstract

One of the primary tasks for effective disaster relief after a catastrophic earthquake is robust communication. In this paper, we propose a simple logistic method based on two-parameter sets of geology and building structure for the failure prediction of the base stations in post-earthquake. Using the post-earthquake base station data in Sichuan, China, the prediction results are 96.7% and 90% for the two-parameter sets and all parameter sets, respectively, and 93.3% for the neural network method sets. The results show that the two-parameter method outweighs the whole parameter set logistic method and the neural network prediction and can effectively improve the accuracy of the prediction. The weight parameters of two-parameter set by the actual field data significantly show that the failure of base stations after earthquake is mainly due to the geological differences where the base stations are located. It can be envisioned that if the geological distribution between the earthquake source and the base station is parameterized, the multi-parameter sets logistic method can not only effectively solve the failure prediction after earthquakes and the evaluation of communication base stations under complex conditions, but also provide site selection evaluation for the construction of civil buildings and power grid towers in earthquake-prone areas.

## Introduction

Earthquake disasters can cause collapse of houses, damage to communication base stations towers and transmission lines, resulting in the disruption of communication services over a large area. The interruption of communication services prevents effective information exchange in the affected areas, which seriously hinders rescue and causes huge loss of life and property to local people. How to ensure communication after the earthquake is a key concern for disaster prevention and mitigation. In order to grasp the operation condition of post-earthquake communication base stations, Liu et al.^1^ from China Earthquake Administration conducted a study and analysis of typical seismic damage of Wenchuan post-earthquake ground base stations and floor base stations to obtain the operational factors affecting post-earthquake communication base stations, respectively. Bai et al.^2^ used a fault tree model to analyze the causes of service withdrawal of base station due to earthquake damage and obtained the failure probability of post-earthquake communication base stations. Li et al.^3^ derived the failure probability of post-earthquake communication base station rooms and towers and gave an assessment by conducting seismic vulnerability analysis of ground base station equipment and facilities. Thakur et al.^4^ selected rooftop towers of buildings with different slopes for dynamic analysis and comparison. Masoud et al.^5^ analyzed the seismic damage probability and vulnerability assessment of combined steel–concrete structures using finite element software. Agrawal et al.^6^ combined stochastic factors and real data to simulate earthquake occurrence and proposed a stochastic model to estimate the impact of seismic hazard on backbone optical network. To provide communication services to post-earthquake disaster areas, Peer et al.^7^ proposed a new multi-hop device-to-device (D2D) communication framework that connects devices which are not in the coverage area of the communication network to active base stations through relaying. Ibrah et al.^8^ designed a scheme to build an emergency relay network using multiple UAVs. Yao et al.^9^ designed an emergency wireless communication resource allocation scheme based on 5G-UAV. Li et al.^10^ proposed an air-ground integrated “Super-BTS” to form a dual routed transmission of local ring network + satellite transmission.

The above studies mainly analyzed the causes of failures based on the working conditions of post-earthquake communication base stations or propose a new emergency communication scheme. These new solutions are difficult and costly to implement in practice and are not conducive to post-earthquake disaster relief work. The post-earthquake communication base station condition analysis is limited to the relationship between the tower type of the base station^11^, building structure^12^, etc. and the earthquake. While ignoring that the damage of the post-earthquake communication base station is also related to many factors such as the geographical location of the base station, the distance from the earthquake source, the geography and geology between the earthquake source and the communication base station. Through the analysis of the causal relationship between the post-earthquake communication base station working conditions and these factors, a reasonable model is selected to make the siting of communication base stations in catastrophic earthquake-prone areas reliable, thus effectively reducing the failure rate of post-earthquake communication base stations. At present, there is no reliable method for siting and constructing communication base stations in earthquake-prone areas, and they are implemented according to standards, such as selecting seismic intensity according to geographical areas, there will be problems such as increased investment and failure of communication base stations after earthquakes. In fact, the sitting of communication base stations requires a specific analysis process, so that the causes of post-earthquake failure problems can be known, and relevant preparations can be made at the communication base station sitting stage.

Based on the real operation data of post-earthquake communication base stations, this paper proposes a logistic method of parameter grouping, which can effectively evaluate the failure probability of post-earthquake communication base stations. And it can be used to judge whether the location of communication base stations is proper, to reduce the probability of failure of post-earthquake communication base stations, so that failure-free base stations can form the continuous coverage of emergency communication networks, and thus reducing the special investment in post-earthquake emergency networks.

## Conclusion

In the past, it was often thought that complex nonlinear methods such as artificial intelligence were needed to solve the problem of accurate prediction and evaluation of the working conditions of communication base stations after earthquakes, but in fact, traditional simple methods can improve the accuracy of prediction and evaluation just as well when more factors such as geology and geography and building structure are taken all into account. The advantage of traditional Logistic method compared to the method of innovative technology such as artificial intelligence is that the whole physical process is clear. Through the judgment of weighed-parameters of two-parameter set, it is derived clearly that geological factors have the greatest impact on communication base stations after earthquakes. In the construction of base stations, we can effectively reduce the construction cost of base stations by selecting the location of base stations reasonably rather than seismic intensity level.

### Data processing and discussion

#### Data sources

**Figure 1:**
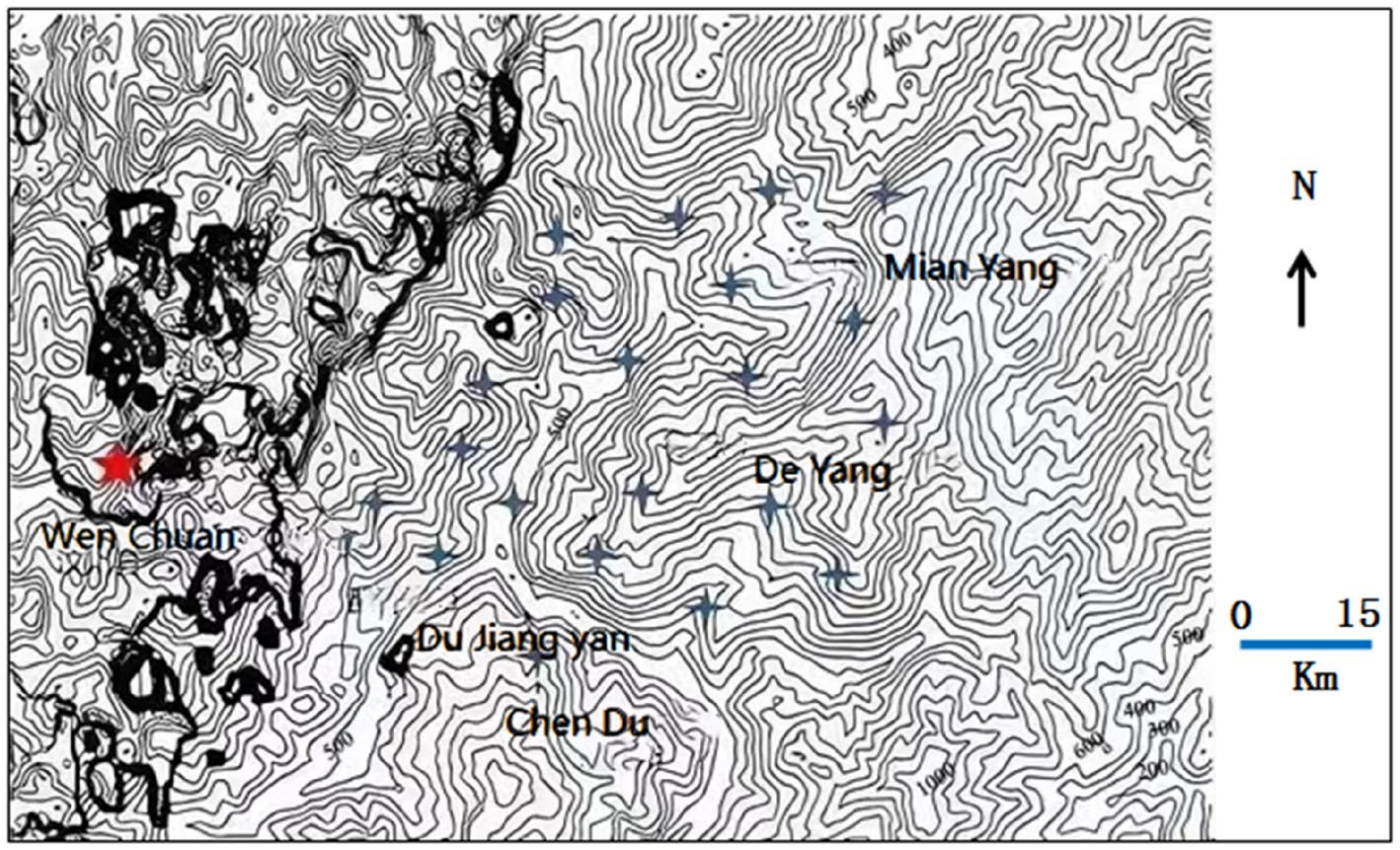
Wenchuan Ms8.0 earthquake.

**Figure 2:**
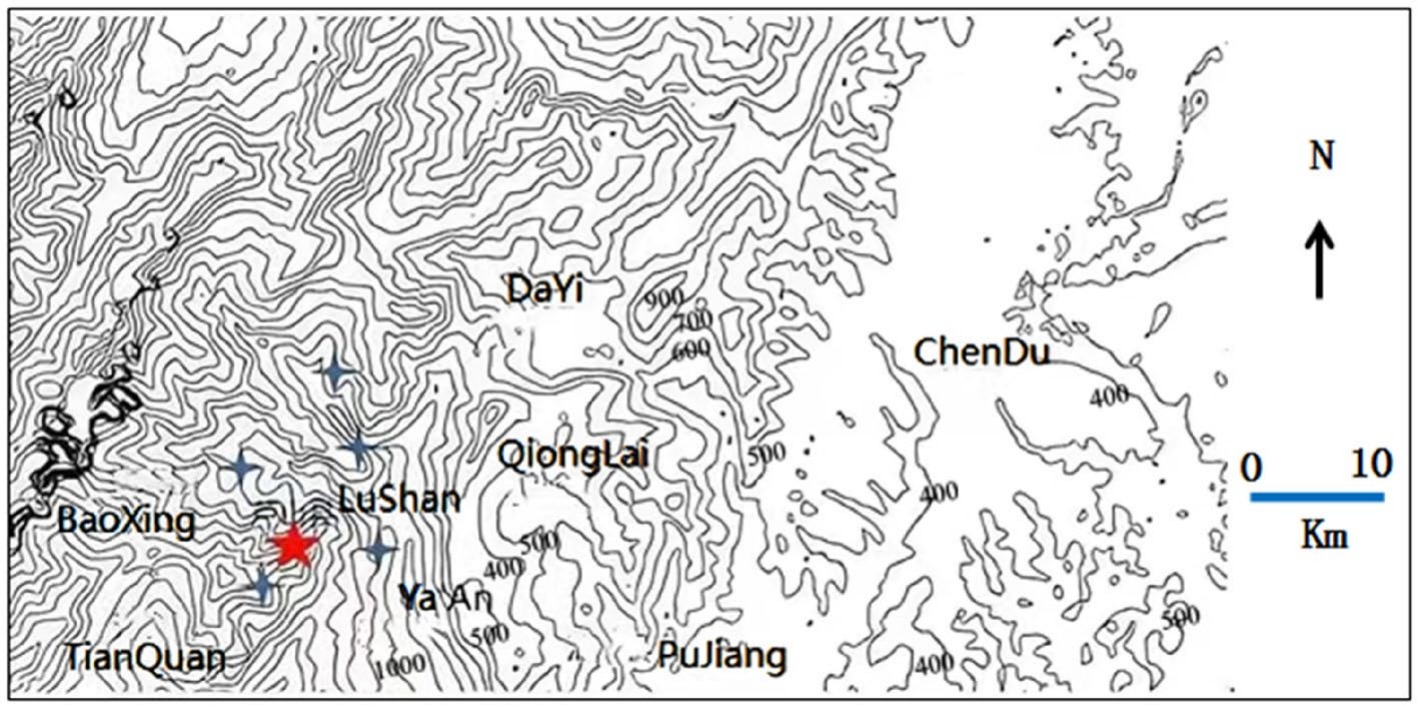
Lushan Ms7.0 earthquake.

**Figure 3:**
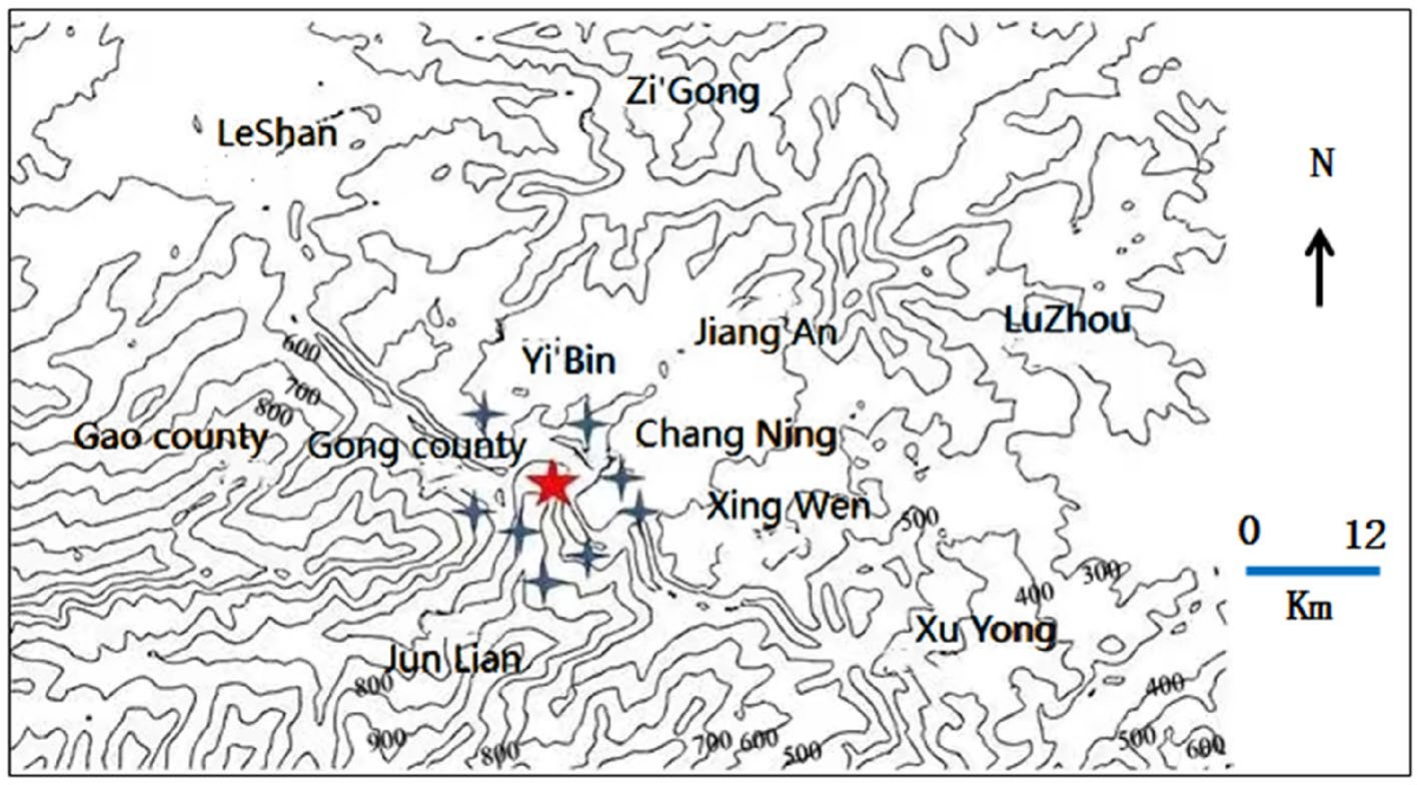
Changning Ms6.0 earthquake.

To avoid the accidental source of post-earthquake communication base station data in a single region, it is necessary to select post-earthquake communication base station data from different earthquake magnitudes, different earthquake fault zones, different geological and geographical environments, and other composite factors when selecting experimental data. In this paper, the post-earthquake communication base station statistics of three earthquakes, Wenchuan Ms8.0 earthquake, Lushan Ms7.0 earthquake and Changning Ms6.0 earthquake in Sichuan Province, are used as the object of analysis and research. The geographical contour maps of the seismic regions are shown in Figs. 1, 2 and 3, respectively. The pentagram is the epicenter, and the quadrangular star is the cities and counties where the base station is distributed.

Figure 1 shows that the selected base station data are mainly distributed in Dujiangyan City, Deyang City, Mianyang City, and Wenchuan County. These areas have dense geographical contours, are located in the northeast of Chengdu city, and are distributed along the Longmenshan earthquake fault zone; the fault zone is northeast-southwest trending, the topography is mainly mountainous and hilly, the geological structure is more complex, and a variety of rock formations coexist, and the communication base stations are mostly mountain base stations dominated by ground stations.

Lushan County is a district and county under Ya’an City. As can be seen in Fig. 2, Lushan County is next to Chengdu City in the east and Wenchuan County in the north and is in the southern part of the Longmenshan earthquake fault zone, with the whole territory being a basic intensity 7 degrees or higher intensity zone. The topography is high in the north and low in the south, there are more mountain valleys in the county, and the geological structure is mainly sedimentary rock, and communication base stations are mainly mountain base stations.

Changning is a county of Yibin, located in the southeast of Yibin City and belongs to Huayingshan fault zone. As can be seen from Fig. 3, the terrain is high in the South and low in the north, mainly mountainous and hilly, with complex geological structure and mainly sedimentary rocks.

#### Parameter selection

Although all three earthquakes occurred in Sichuan Province, China, they were in different areas with different magnitudes and seismic intensities. In the post-earthquake survey, it was found that the communication base stations could maintain basic operation if the main equipment such as power supply system, wireless equipment and transmission equipment were not damaged after the earthquake, and the towers played the role of antenna support and information transmission without changes; if any of them were damaged, the communication base stations could not operate effectively^3^. Therefore, scenarios can be classified based on the above description of the post-earthquake base station room condition and tower condition, normal or damaged is selected as the measurement criteria.

The impact of earthquakes on communication base stations can be divided into geological factors underground and environmental factors above ground. The data in this article is from Sichuan Province, China. Sichuan Province is a mountainous area, and the lithology of the area is mainly sedimentary, magmatic and metamorphic rocks, so we use these three rocks as a description of geological structure. The above-ground environmental factors include the geographical location of the communication base station construction and the construction type of the communication base station^3,4,13,14^. The occurrence of earthquakes is on seismic fault zones, which are directional in nature, and the communication base stations are more severely damaged in the area towards the seismic fault zone, and the seismic intensity is higher in this direction, which requires the selection of the Base station relative epicentral position in the selection of parameters.

According to the above definition of variable parameters that affect the working conditions of the base station, the parameters can be divided into local variable parameters and global variable parameters. Global variables have global scope, which means that all communication base stations in the affected area are affected by the same objective factors at the time of an earthquake, such as magnitude, seismic intensity, geological structure, and the relative orientation of the base station to the epicenter. Local variables only have a local scope, which are affected by the geological and geographical characteristics of the communication base station location, the structure of the base station and other factors, such as magnitude, seismic intensity, geological structure, geographical location of the base station, base station category, computer room situation, tower type, etc. Earthquake magnitude and seismic intensity are the direct factors affecting the working conditions of post-earthquake communication base stations, so the two divisions of local variable parameters and global variable parameters overlap in magnitude and seismic intensity.

Due to the enormous number of influencing factors, the small amount of data, and the significant differences between local factors such as geological changes and seismic grade, a two-parameter set consisting of global and local parameters was modeled, and the results were derived from the weighted sum of the local and global parameters estimated through binary regression. The local parameters and global parameters are shown in Tables 1 and 2, respectively. The factors affecting the base station operation are set as input variables *X*, and whether the base station operates after the earthquake is set as output variable *Y*.

**Table 1:** Local variable parameters.

Variable	Variable parameters
Magnitude ${{\varvec{X}}}_{1}$	6.0, 7.0, 8.0
Seismic intensity ${{\varvec{X}}}_{2}$	6, 7, 8, 9, 10, 11
Geological structure ${{\varvec{X}}}_{3}$	Sedimentary rock, magmatic rock, metamorphic rock
Base station location ${{\varvec{X}}}_{4}$	Flat ground, Half-hillside, mountaintop
Base station category ${{\varvec{X}}}_{5}$	Ground base station, rooftop base station
Base station room condition ${{\varvec{X}}}_{6}$	Normal, damaged
Tower condition ${{\varvec{X}}}_{7}$	Normal, damaged

**Table 2:** Global variable parameters.

Variable	Variable parameters
Magnitude ${{\varvec{X}}}_{1}$	6.0, 7.0, 8.0
Seismic intensity ${{\varvec{X}}}_{2}$	6, 7, 8, 9, 10, 11
Geological structure ${{\varvec{X}}}_{3}$	Sedimentary rock, magmatic rock, metamorphic rock
Base station relative epicentral position ${{\varvec{X}}}_{8}$	Due east, northeast, due north, northwest, due west, southwest, due south, southeast

#### Data pre-processing

If collinearity occurs in the quantified data, it will seriously affect the inference and prediction in regression analysis. In this paper, the variance inflation factor *VIF* is used to detect covariance in the data^15^. When 0 < *VIF* < 10, there is no collinearity. The *VIF* value between the actual data is less than 2, there is no collinearity in the data set. The results are shown in Table 3.

**Table 3:** VIF value.

Variable	${{\varvec{X}}}_{1}$	${{\varvec{X}}}_{2}$	${{\varvec{X}}}_{3}$	${{\varvec{X}}}_{4}$	${{\varvec{X}}}_{5}$	${{\varvec{X}}}_{6}$	${{\varvec{X}}}_{7}$	${{\varvec{X}}}_{8}$
*VIF*	1.57	1.59	1.71	1.63	1.82	1.25	1.33	1.25

**Figure 4:**
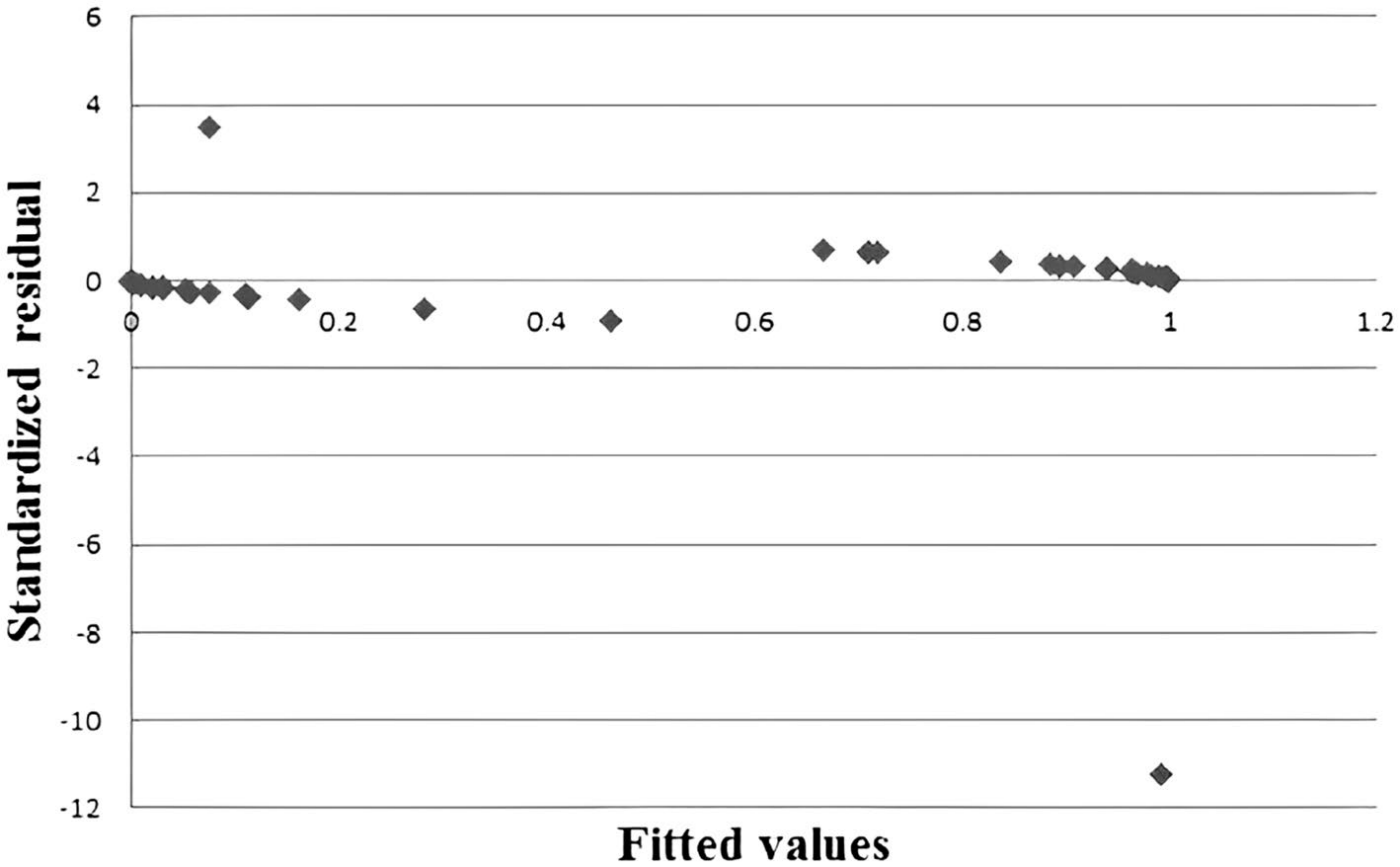
Outlier detection results.

To avoid outliers in the data set from affecting the results of regression analysis, standardized residuals were used for testing^15^. There are two sets of experimental data outliers on the 95% confidence interval, and the outlier data were removed. The results are shown in Fig. 4.

The problem of different nature and comparability of data is solved by using data normalization to unify the comprehensive variables to the same standard through homogenization and dimensionless processing of multiple variables parameters in the data set after eliminating outliers. In this paper, 80 sets of experimental data were finally selected and randomly divided into training and test sets in the ratio of 7:3 for experiments.

### Experimental results and discussion

In this subsection, the logistic model and the two-parameter set model are used to predict the working condition of the post-earthquake base station respectively. In view of the dynamic changes in the location of the earthquake source and the operating working condition of the communication base station, a neural network model^16–20^ with an autonomous learning function that can find the optimal solution at high speed by continuous feature learning in the face of complex environmental problems is also introduced for comparison. The neural network model in this paper is a three-layer structure, with the first layer as the input layer, taking the variable parameters as the input; the second layer as the hidden layer, responsible for weighting the data; and the third layer as the binary output layer, deciding the base station operating conditions based on the output. The coefficients of the hidden layer of the neural network are adjusted by the backward propagation algorithm to complete the model training.

In the experiment, the default classification threshold of the binary classification model is 0.5, which is not reasonable in specific scenarios. In this paper, the method is applied to practical engineering, and the threshold value is selected according to the maximum prediction correct rate in the training set results. The distribution of the prediction probability of post-earthquake communication base station conditions in the training set is analyzed; the correct prediction rate is the maximum when the threshold is set to 0.4. According to the training set training results, the result is judged as 1 when the output prediction probability value* P* > 0.4 and 0 when *P* < 0.4.

Table 4 shows the correct prediction rate of the logistic model with a threshold value of 0.4 compared with the default threshold value of 0.5, which is used to demonstrate that a threshold value of 0.4 is more suitable for the model in this paper than a threshold value of 0.5. The correct prediction rate in the training set is 86.00% when the threshold is 0.5 and 88.00% when the threshold is 0.4. The correct prediction rate in the test set is 86.70% when the threshold is 0.5 and 90.00% when the threshold is 0.4. With reasonable threshold choice, the correct prediction rates of the training and test sets are improved by 2% and 3.3%, respectively. Therefore, in the sense of probability distribution, it is more reasonable for the model to set the threshold value to 0.4. Subsequent experiments on the two-parameter set model and the neural network model are based on the threshold value of 0.4.

**Table 4:** Threshold selection.

Sample	Threshold	Actual	Predicted results		Accuracy rate (%)
			0	1	
Training	0.5	0	19	2	86.00
		1	5	24	
	0.4	0	19	2	88.00
		1	4	25	
Test	0.5	0	13	1	86.70
		1	3	13	
	0.4	0	13	1	90.00
		1	2	14	

Table 5 gives a comparison of the correct prediction rates for three groups of models including the two-parameter set model. Among them, the logistic method and the neural network method use non-group parameter experiments, the two-parameter set model based on the logistic method uses grouping parameter experiments with grouping parameter weights of *a* and *b*, where *a* is the local parameter weight and *b* is the global parameter weight.

**Table 5:** Experimental results.

Models	Actual	Predicted results		Accuracy rate (%)
		0	1	
Logistic	0	13	2	90
	1	1	14	
Two-parameter	0	14	0	96.7
	1	1	15	
Neural network	0	12	1	93.3
	1	1	16	

Several factors affecting the post-earthquake communication base station operating conditions are modeled with non-grouping and grouping parameters, respectively. Based on the grouping results, the probability weighted accumulation obtained by conducting evaluation prediction using the two classification parameters obtained from regression modeling is jointly evaluated to predict the post-earthquake base station operating conditions. As shown in Table 5, among the three groups of models, the prediction correctness of the two-parameter model using grouping is perfectly accurate and much higher than that of the non-grouping logistic method and neural network model.

The method of parameter grouping can clearly present the relationship between the grouped parameters. The weight of the global parameter obtained from the field data processing is 2.2 times of the weight of the local parameter, showing that the influence of the geological parameter and the base station relative epicentral position in the global parameter is huge, showing that the choice of the base station construction related to geological factors is important. The non-grouping parameters do not reflect the role played by a specific parameter among the influencing factors, while in the grouping parameters we can indicate which parameter is more important by the weights. By this method it is possible to consider geology as the first factor in the failure prediction of post-earthquake communication base stations or in the choice of future base station construction sites.

In this study, we found that in addition to the traditionally perceived factors such as magnitude and seismic intensity, base station relative epicentral position and geological geography are also factors that affect the post-earthquake communication base station working conditions, among which geological factors are the most important. Unlike the previous methods for predicting the post-earthquake base station working conditions, the two-parameter method of grouping these influencing factors can extract useful information of the influencing factors to a greater extent and improve the correct prediction rate in a complex geographical background environment using a smaller number of base stations.

## Methods

The operating condition of a post-earthquake base station can be represented by a binary classification, i.e., 1 is operation and 0 is fault, so the prediction and evaluation of its operating condition is a binary classification problem. Therefore, a binary classification (0 or 1) method is needed to estimate the operating condition of the post-earthquake base station. Logistic regression is a method based on linear regression theory, which can effectively deal with the binary classification (0 or 1) problem with distinct characteristics by introducing nonlinear factors through the sigmoid function. It is often used in data mining, automatic disease diagnosis, economic forecasting and other fields^21–25^.

The essence of logistic regression is to divide the probability of occurrence by the probability of non-occurrence, and then take logarithm, so that a linear relationship is formed between dependent variables and independent variables. The logistic model is set up as follows:

1 $$\mathrm{ln}(\frac{P}{1-P}) = {\beta }_{0}+{\upbeta }_{1}{X}_{1}+{\upbeta }_{2}{X}_{2}+\dots +{\beta }_{i}{X}_{i}$$

The above formula can visually present the probability of dependent variable after transformation:

2 $$\mathrm{P}=\frac{1}{1+{e}^{-({\beta }_{0}+{\upbeta }_{1}{X}_{1}+\dots +{\beta }_{i}{x}_{i})}}$$

where P is the probability of post-earthquake communication base stations; ${\beta }_{0}$ is a constant term, ${\beta }_{1},{\beta }_{2},\dots ,{\beta }_{i}(i\ge 1,i\in \gamma )$ is an independent variable parameter, ${X}_{1},{X}_{2},\dots ,{X}_{i}(i\ge 1,i\in \delta )$ is an independent variable.

**Figure 5:**
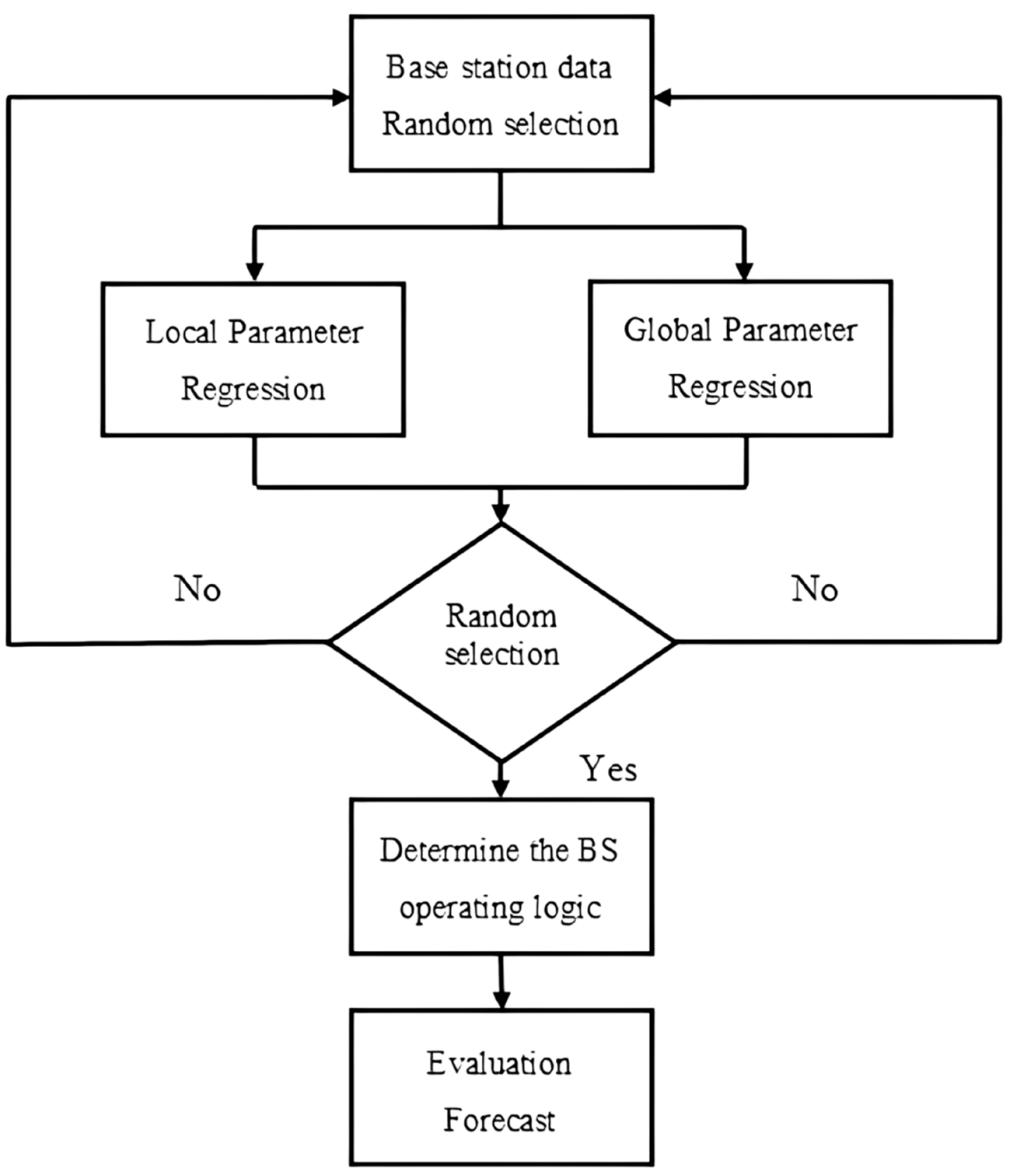
Logic diagram for determining the working conditions of communication base stations.

Due to the limited actual data of post-earthquake communication base stations, the amount of data in this paper has just reached the requirement of logistic regression analysis, i.e., the amount of data is 10 times of the variable parameters. It is unreasonable to process the regression parameters by data from only a single regional earthquake source, and the accuracy of the obtained results may not be high, so it is necessary to analyze and figure out the distribution of regression parameters using random combinations of data from different earthquake sources. To avoid the low prediction accuracy caused by the small amount of data, this study proposes a two-parameter set method based on logistic regression to improve the prediction accuracy, and the processing flow of the post-earthquake base station failure probability with two-parameter set is given in Fig. 5.

### Data grouping

The communication base station data from different seismic sources are randomly combined and randomly divided into training set and test set according to the ratio of 7:3. 70% of the training set data are used for learning and 30% of the test set data are used for testing.

### Two-parameter regression

Step 1, the first set of base station data is randomly selected in the training set, and the base station data is divided into the set of global variable parameters and local variable parameters. Perform logistic regression tests on these two parameters sets to obtain the prediction probabilities of the global variable parameters and the prediction probabilities of the local variable parameters.

Step 2, the second set of base station data is randomly selected in the training set, repeat the first step, …, until the logistic parameter regression test is completed for all finite data combinations, and obtain the prediction probabilities of all global variable parameters and local variable parameters in the training set.

### Two-parameter weights

The weights are obtained by using the post-earthquake base station conditions of the test data, giving the post-earthquake base station conditions as *Z*, and the post-earthquake base station conditions of normal and failure as 1 or 0, respectively, with the weights set to* a* and *b*, and the prediction probabilities obtained from the local and global parameters of the model are set to *x* and *y*, respectively, *c* as constants. One communication base station corresponds to a binary equation, and the equation for post-earthquake communication base station condition prediction is set up according to the number of actual test data, the weights *a* and *b* can be obtained by the principal component analysis method^15^, see Eq. (3).

3 $$Z=ax+by+c$$

The weights *a* and *b* were obtained using principal component analysis in three steps:

The first step is to determine the composite score of local parameters. Firstly, the selected local variable parameters are subjected to principal component analysis to obtain the corresponding principal components. Then, the composite score model is calculated from the variable coefficients, component contribution rates and cumulative contribution rates of the principal components, and finally, the selected local parameter data are brought into the above local composite score model to obtain the composite score of the base station data, which is denoted as* k*.

The second step determines the composite score of global parameters. In the same way as the local parameter weights are decided; the composite score of the base station data is calculated and denoted as *g*.

The third step is to determine the weight. Using the *k* derived in the first step and the *g* derived in the second step, the weights *a* and *b* are calculated for the local and global parameters, respectively.

Based on the grouped prediction probabilities from the two-parameter group regression, a weighted accumulation of grouped probability values is performed to obtain the final prediction evaluation probabilities^24^.

Through the above learning process, the weight parameters of the model can be obtained, and the weight parameters of the model can be used to predict the post-earthquake conditions of 30% of the test set of base station data once they are determined.

In this method, the geological structure, geographic location of the base station, and the category of the base station in the parameter variables are objectively available when evaluating the siting of communication base stations in earthquake-prone areas. Base station relative epicentral position can be determined according to the preset epicenter, the earthquake magnitude and seismic intensity are preset according to human needs.

However, the condition of the base station room and the tower, which are also input parameters, are unknown. The joint solution of seismic magnitude and seismic intensity is used to judge the seismic capability of the communication base station infrastructure. The logic given to the post-earthquake condition of the communication base station infrastructure is H, and its normal and failure are 1 or 0, respectively. The weights of seismic intensity and magnitude are set to *m* and *n*, respectively, and seismic intensity and magnitude are set to $\mathrm{\alpha }$ and $\upbeta$, respectively, with *e* as a constant.

A binary linear system of equations is set up, with ${\mathrm{H}}_{1}$ as the fortification value and ${\mathrm{H}}_{2}$ as the preset value. Calculate the fortification value and the preset value according to Eq. (4), and then compare the two values. If ${\mathrm{H}}_{1}$ is greater than ${\mathrm{H}}_{2}$, the input is 1, which means that the base station room and tower are in normal condition after the earthquake; if ${\mathrm{H}}_{1}$ is less than ${\mathrm{H}}_{2}$, the input is 0, which means that the base station room and tower fail after the earthquake. By comparing ${\mathrm{H}}_{1}$ and ${\mathrm{H}}_{2}$, we can decide the condition of the existing base stations and towers after the earthquake.

4 $$\left\{\begin{array}{c}{H}_{1}=m{\alpha }_{1}+n{\beta }_{1}+{e}_{1}\\ {H}_{2}=m{\alpha }_{2}+n{\beta }_{2}+{e}_{2}\end{array}\right.$$

## Data Availability

The datasets used during the current study available from the corresponding author on reasonable request.
